# Small Pox and Vaccination

**Published:** 1853-10

**Authors:** 


*Small Pox and Vaccination.*

In the London Lancet for September we find a review of the report of the Small-Pox and Vaccination Hospital, London.

The subjects discussed are:

1. Natural Small-pox.
2. Small-pox after small-pox.
3. Small-pox after vaccination.

The conclusions of the author of the report are as follows:

"1st. That the natural small-pox destroyed about one-third of all whom it attacked.

2nd. That small-pox after small-pox was of comparatively rare occurrence; that a second attack of natural small-pox was rare, but not often fatal, and that protection seemed to be the law. That after inoculated small-pox an attack of small-pox had more frequently led to fatal results; but there is reason to presume that the virus used for inoculation, like a great deal of the lymph used at the present day for vaccination, was often taken at too advanced a period of the disease, and thus did not afford the full measure of protection it was capable of affording if taken at a proper time.

3rd. That vaccination performed in infancy afforded almost complete protection against the fatality of small-pox to the period of puberty; that a variety of circumstances conspired to make it almost impossible to ascertain exactly in what proportion to the vaccinated cases of small-pox subsequently occurred, or might occur, if all persons lived to an advanced age.

4th. That as a matter of safety it would be well for all persons who were vaccinated in infancy to be re-vaccinated at puberty; this measure being more especially requisite for those who were either indifferently or doubtfully vaccinated in infancy, and still more necessary for those who, though vaccinated, had no cicatrix remaining. Finally, as a matter of precaution, it would be desirable that all persons should be re-vaccinated on small-pox existing in the house where they were residing—a precaution, however, that will cease to be necessary to advise when all persons have the benefit of proper and efficient vaccination."

